# The Dual Nature of Onuf’s Nucleus: Neuroanatomical Features and Peculiarities, in Health and Disease

**DOI:** 10.3389/fnana.2020.572013

**Published:** 2020-09-04

**Authors:** Roberta Schellino, Marina Boido, Alessandro Vercelli

**Affiliations:** ^1^Department of Neuroscience Rita Levi Montalcini, University of Turin, Turin, Italy; ^2^Neuroscience Institute Cavalieri Ottolenghi (NICO), University of Turin, Turin, Italy; ^3^National Institute of Neuroscience, Turin, Italy

**Keywords:** aging, autonomous nervous system, injury, motor neuron diseases, neurodegeneration, somatic motor neurons, spinal cord

## Abstract

Onuf’s nucleus is a small group of neurons located in the ventral horns of the sacral spinal cord. The motor neurons (MNs) of Onuf’s nucleus innervate striated voluntary muscles of the pelvic floor and are histologically and biochemically comparable to the other somatic spinal MNs. However, curiously, these neurons also show some autonomic-like features as, for instance, they receive a strong peptidergic innervation. The review provides an overview of the histological, biochemical, metabolic, and gene expression peculiarities of Onuf’s nucleus. Moreover, it describes the aging-related pathologies as well as several traumatic and neurodegenerative disorders in which its neurons are involved: indeed, Onuf’s nucleus is affected in Parkinson’s disease (PD) and Shy-Drager Syndrome (SDS), whereas it is spared in Amyotrophic Lateral Sclerosis (ALS), Spinal Muscular Atrophy (SMA), Duchenne Muscular Dystrophy (DMD). We summarize here the milestone studies that have contributed to clarifying the nature of Onuf’s neurons and in understanding what makes them either vulnerable or resistant to damage. Altogether, these works can offer the possibility to develop new therapeutic strategies for counteracting neurodegeneration.

## Introduction

In 1899, for the first time, the Russian neuroanatomist Bronislaw Onuf (Onufrowicz) identified and described a group of neurons located in the human sacral spinal cord and involved in the innervation of the pelvic floor: he called this formation “nucleus X,” later renamed after him “Onuf’s nucleus.”

**Figure 1:**
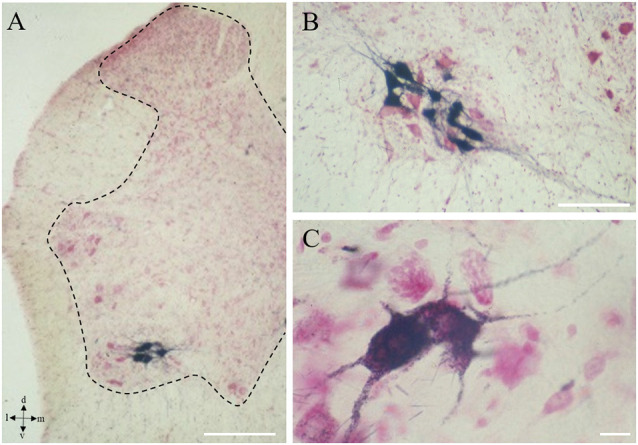
Onuf’s nucleus motor neurons (MNs). Localization of Onuf’s MNs (in black those innervating ischiocavernosus muscle, in the dorsolateral column) in rat spinal cord, by horseradish-peroxidase retrograde transport, at low **(A)** and high magnification **(B,C)**, in a neutral red-stained section. Dendrites form characteristic dendritic bundles. The dotted line in panel **(A)** outlines the gray matter. Scale bars: 100 μm in panels **(A,B)**, 10 μm in panel **(C)**.

Onuf’s nucleus (Figure 1) has been identified in different locations through the species, similar in the cat, dog, monkey, and man, differently from the rat, Mongolian gerbil, and domestic pig (as reviewed by Gerrits et al., 1997). The human Onuf’s nucleus is located in the ventral horn of the upper sacral segment including S2–S3 and rarely also involving S1 (with an overall length comprised between 4 and 7 mm). In 1981, Schrøder (1981) described three portions of human Onuf’s nucleus, distinguishable based on their cytoarchitecture: cranial, dorsomedial and ventrolateral. Later on, Forger and Breedlove only confirmed the presence of the dorsomedial and ventrolateral cell groups in humans (Forger and Breedlove, 1986), and this subdivision is generally still used.

**Figure 2:**
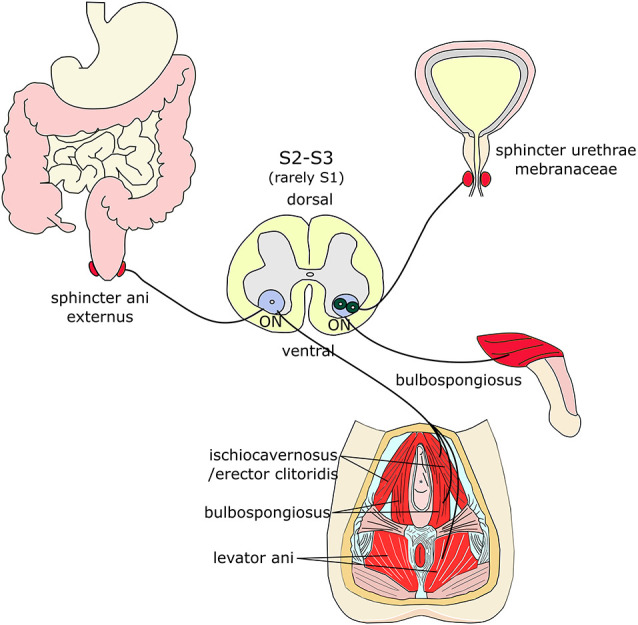
Muscles innervated by Onuf’s MNs. Onuf’s (ON) MNs are located in the ventral horns of the upper sacral segment and innervate sphincters and muscles of the pelvic and perineal regions, such as the sphincter ani externus, the sphincter urethrae membranaceae, the bulbospongiosus, the ischiocavernosus (and its equivalent in females, erector clitoridis), and the levator ani muscles.

Onuf’s nucleus innervates voluntary striated muscles, and its MNs share several morphological aspects with other somatic MNs. However, its rich peptidergic innervation suggests its autonomic involvement (Kawatani et al., 1986; Pullen et al., 1997). In 1900, Onuf and colleagues (Onuf, 1900) reported that it innervates the only ischiocavernosus (and it’s equivalent in females, erector clitoridis) and bulbospongiosus (or bulbocavernosus) muscles. However, later on, other authors clarified that Onuf’s nucleus also controls the sphincter urethrae membranaceous muscle (external urethral sphincter muscle, EUS), the levator ani (elevator muscle of the anus), and the sphincter ani externus (external anal sphincter, EAS; Gerrits et al., 1997; reviewed by Mannen, 2000; Figure 2). Therefore, through the control of the pelvic-perineal muscles, it plays a crucial role in erection and urinary/fecal continence and participates in ejaculation. Moreover, some authors have also hypothesized a somatotopic arrangement in the Onuf’s nucleus (both in humans and in animals), suggesting that MNs of the dorsomedial portion innervate the EAS, while those of the ventrolateral portion innervate the EUS (Iwata et al., 1993).

**Figure 3:**
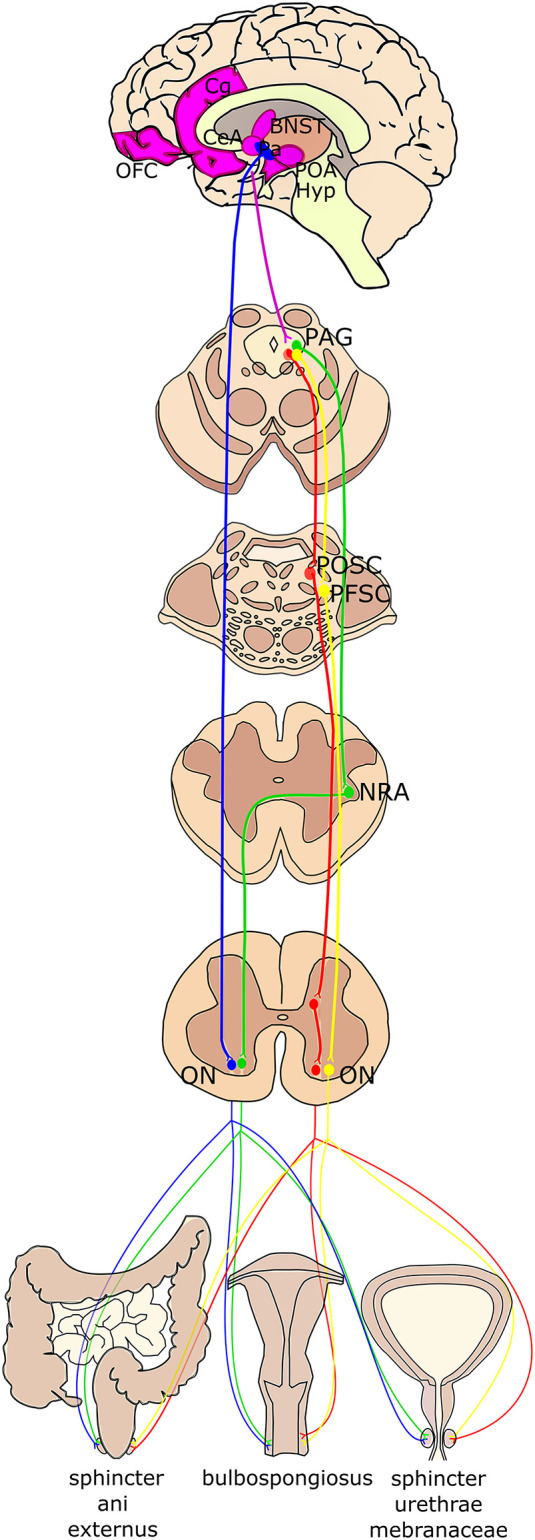
Schematic summary of the higher-order projections to Onuf’s nucleus. Onuf’s nucleus (ON) receives direct projections from the paraventricular hypothalamic nucleus (Pa; in blue). The periaqueductal gray (PAG) in the mesencephalon controls the pelvic floor by stimulating the pelvic organ stimulating center (POSC; in red), which in turn projects to inhibitory interneurons in the sacral cord that finally innervate Onuf’s nucleus. Moreover, PAG sends efferent fibers to the nucleus retroambiguus (NRA; in green) in the medulla, which is then connected with Onuf’s MNs, which innervate the muscles determining the abdominal pressure. The strongest afferents to the PAG originate from different brain areas (identified in purple), such as the medial orbitofrontal cortex (OFC), the anterior cingulate gyrus (Cg), the preoptic area and hypothalamus (POA, Hyp), the lateral Bed Nucleus of Stria Terminalis (BNST) and the Central Nucleus of the Amygdala (CeA). Finally, projections from the limbic system and prefrontal cortex reach the PAG that then projects to the pelvic floor stimulating center (PFSC; in yellow) in the pons, which in turn innervates Onuf’s MNs.

In 1987, Holstege and Tan traced Onuf’s nucleus afferent fibers, by using HRP and Tritiated L-Leucine labeling: they demonstrated a direct connection with the brainstem and hypothalamus (Holstege and Tan, 1987), therefore confirming the contribution of Onuf’s nucleus to the basic physiologic functions regulated by these brain regions. Interestingly, since the “80s, direct and specific subcortical projections to Onuf”s MNs were shown to originate from both diencephalon and brainstem (Figure 3), including: (i) the ipsilateral paraventricular hypothalamic nucleus; (ii) the ipsilateral caudal pontine lateral reticular formation; and (iii) the contralateral caudal nucleus retroambiguus (NRA; Holstege and Tan, 1987). Indeed, as demonstrated in rats by transneuronal virus tracing methods, many neuronal populations of the CNS are involved in the supraspinal control of micturition, defecation and sexual functions (Fowler et al., 2008): some spinal projections are aspecific diffuse (such as the noradrenergic cells of the locus coeruleus, the serotoninergic neurons of the medullary raphe nucleus and the A5 noradrenergic cell group proximal to the superior olivary complex in the pontine tegmentum; Fowler et al., 2008; de Groat, 2017), whereas others specifically regulate pelvic organ function. These latter include the neurons of pelvic organ stimulating center (POSC), also known as Barrington’s nucleus or pontine micturition center, in the rostral pontine tegmental region, which represents the unique and specific projection from the brainstem to the sacral MNs (Fowler et al., 2008; Holstege, 2016). POSC maintains direct projections through the entire length of the spinal cord to the parasympathetic MNs in the sacral segments (Holstege, 2016), controlling the detrusor muscle of the bladder and other pelvic organs (Fowler et al., 2008). Micturition control is also strictly related to emotions, therefore requiring the involvement of the limbic system. Indeed many brain regions (taking part in or connected to the limbic system) have the important role to verify the safety of the environment where the micturition takes place, eventually preventing it when the optimal conditions are not guaranteed, even if signals to urgently empty the bladder come from the sacral cord. This conflict can strongly influence human social behavior, causing embarrassing related to wetting pants, both in childhood and in the elderly (for an extensive review see Holstege, 2005).

Moreover, collateral fibers of POSC have synapse to the inhibitory premotor interneurons in the spinal cord that are connected with Onuf’s MNs, so that contraction of the bladder, rectum, and uterus occurs together with the relaxation of the pelvic floor striated muscles, which is physiologically important for micturition, defecation and delivery, respectively (Fowler et al., 2008; Holstege, 2016). Tracers injected into specific hypothalamic nuclei were anterogradely transported to the terminals reaching the spinal nuclei involved in pelvic functions (de Groat, 2017): indeed, tracers into the paraventricular nucleus reached the sacral parasympathetic nucleus as well as the Onuf’s nucleus (Argiolas and Melis, 2005). Moreover, the preoptic area and the lateral hypothalamus send projections to POSC (Kuipers et al., 2006; Holstege, 2016). Neurons in the POSC in turn project primarily to the sacral parasympathetic nucleus, the lateral edge of the dorsal horn, and the dorsal commissure. Conversely, projections from neurons in the lateral pons, a region involved in the control of the urethral sphincter, terminate rather selectively in the Onuf’s nucleus (de Groat, 2017).

However, the strongest afferent projections of the POSC originate from the periaqueductal gray (PAG) in the mesencephalon, which also projects to the pelvic floor stimulating center (PFSC). For instance, once that the PFSC sends inputs to void the bladder, PAG activates the POSC neurons, whose efferent fibers directly activate the parasympathetic preganglionic neurons in the intermediolateral cell column of the sacral spinal cord, leading to detrusor muscle contraction. At the same time, POSC neurons activate GABAergic spinal interneurons leading to Onuf’s nucleus inhibition, which results in relaxation of the striated EUS (Arya and Weissbart, 2017). Interestingly, through the sacral pelvic organ spinal relay center, PAG receives feedback information concerning the urine amount in the bladder and pelvic organ situation, generally *via* long ascending fiber pathways, thus modulating pelvic muscle state through the control of POSC. PAG projects also to the NRA in the ventrolateral tegmentum of the caudal medulla, that, in turns, at least in several mammals (e.g., cats), projects to the MNs of Onuf’s nucleus that innervates the pelvic floor muscles for sexual behavior control (Holstege and Tan, 1987; Holstege, 2016). The NRA projections to the pelvic floor muscles controlled by Onuf’s MNs might be also part of a system that regulates abdominal pressure (Holstege and Tan, 1987). Finally, PAG receives fibers from other brain regions: hypothalamus, preoptic area, amygdala, bed nucleus of the stria terminalis, and the medial orbitofrontal cortex (OFC; Holstege, 2016). These latter projections seem to have a strong impact on the PAG-POSC pathway activation. Indeed, this part of the prefrontal cortex might be considered as the output cortical center, where the activation of POSC and Onuf’s nucleus *via* PAG is decided, to regulate micturition and/or defecation and other sexual behaviors. Interruption of this pathway leads to loss of central control of when to start micturition, resulting in incontinence (Holstege, 2016). Despite the POSC influences subcortical and cortical regions (e.g., hypothalamus, locus coeruleus, PAG, and the medial prefrontal cortex; Michels et al., 2015; Malykhina, 2017) and several micturition centers are located in the cerebral cortex, basal ganglia, and brainstem (Fowler et al., 2008), no direct cortical projections to Onuf’s nuclei have been demonstrated in humans (Iwatsubo et al., 1990; Mannen, 2000); conversely, they were observed in South America monkey Saimiri, in which corticospinal projections start from the tail portion of cortical area 4 and end bilaterally on Onuf’s nuclei (Nakagawa, 1980). Thus, the supraspinal regulation of pelvic tract function depends on multiple paths carrying information between the brain and the spinal cord.

Onuf’s neurons mainly display an ellipsoid shape (44%), and more rarely a circular (37%) or fusiform (19%) shape (Pullen et al., 1997). As the somatic motor neurons (MNs), Onuf’s MNs are multipolar and show large Nissl granules. However, their cell size is smaller compared to the surrounding MNs. Additionally, electron microscopic studies showed that the surrounding neuropil is characterized by few myelinated fibers and numerous vertical unmyelinated fibers arranged as dendritic bundles (Schrøder, 1981; Mannen, 2000): this could explain the appearance of Onuf’s nucleus in a Nissl stain.

Pullen et al. (1997) performed several morphological analyses that allowed defining its dimension and the number and size of neurons. In some cases, these results (although referring to a few subjects and not statistically significant) highlighted a slight sexual dimorphism. For example, the Onuf’s nucleus volume was 0.299 ± 0.0369 mm^3^ in males and 0.285 ± 0.0551 mm^3^ in females; the mean number of MNs was 654 ± 141.8 in males and 610 ± 142.7 in females; on the other hand, the mean neuronal size was very similar between sexes (25.94 ± 3.63 μm in males, and 26.25 ± 2.25 μm in females).

The slight sex dimorphism could depend on androgens: following androgen deprivation, MNs die by apoptosis, whereas the presence of androgens allows MN survival, either primarily or secondarily (i.e., due to muscle atrophy). This could be ascribed to the direct action of androgens on neurons: for example, compared with other striated muscles, MNs of bulbospongiosus muscle can accumulate testosterone (Little et al., 2009) and their target muscles are enriched for androgen receptors in male (Jordan et al., 1997). Notably, androgens can also stimulate muscular fibers to secrete the Ciliary Neurotrophic Factor (CNTF) that retrogradely reaches MN cell bodies and supports MN survival (Forger et al., 1993). Our group also demonstrated the involvement of testosterone: indeed, the prepubertal castration can determine a significant reduction in the somatic and nuclear areas of Onuf’s nucleus MNs, although sparing their number (Vercelli and Cracco, 1990). Additionally, also the neuromuscular endplate area in the ischiocavernosus muscle seems to depend on the presence of testosterone, remaining smaller in castrated rats compared to normal ones (Vercelli and Cracco, 1989).

## Morphological and Neurochemical Properties of Onuf’s Nuclei Neurons

The first morphological/cytochemical studies made difficult the classification of Onuf’s nucleus: indeed, the combination of retrograde tracing with immunocytochemistry staining has initially proposed the Onuf’s nucleus as a somatic nucleus. However, due to its peptidergic inputs, it was later suggested that Onuf’s nucleus shares also autonomic-like features (Kawatani et al., 1986; Katagiri et al., 1988; Tashiro et al., 1989a,b). Interestingly, Onuf’s nucleus is spared in diseases in which somatic sacral MNs degenerate [as Duchenne Muscular Dystrophy (DMD), Spinal Muscular Atrophy (SMA) and Amyotrophic Lateral Sclerosis (ALS)]; on the contrary, the nucleus is affected -together with the parasympathetic nuclei in pathologies in which somatic sacral MNs are spared [such as Shy-Drager Syndrome (SDS), Fabry’s disease, Parkinson’s Disease (PD) and Multiple System Atrophy (MSA)]. The first group of diseases is characterized by somatomotor neuronal disorders, while the second group mainly includes visceromotor neuronal malfunctions. Thus, this characteristic is rather an indication for its dual instead of a unique autonomic nature.

Indeed, Onuf’s nucleus and somatic MNs share some electrophysiological properties. On the other hand, different passive electrical membrane properties have been described, together with low rheobase values (Hochman et al., 1991; Sasaki, 1991); moreover, despite collateral axons are recurrent (Sasaki, 1994), Onuf’s MNs miss recurrent inhibition (Jankowska et al., 1978; Sasaki, 1991).

Thus, this evidence suggests that the real nature of Onuf’s MNs should be investigated biochemically, to better define their specificities and to clarify their involvement in the abovementioned pathologies. The neurochemical properties of Onuf’s neurons have been initially studied since the later ‘80s.

It has been demonstrated, in both human and laboratory animals, that Onuf’s nucleus is densely innervated by CPON- (C-terminal Flanking Peptide of Neuropeptide Y), enkephalin- and somatostatin-immunopositive fibers, suggesting an autonomic nature of these cells. In specific areas of the neuropil, dense aggregates of peptide-immunoreactive fibers are visible in the proximity of Onuf’s nucleus (Gibson et al., 1988; Katagiri et al., 1988; Pullen et al., 1997). Indeed, the density of enkephalin-immunopositive varicosities is higher than over somatic neurons (Ramírez-León et al., 1994), and enkephalin fibers outline Onuf’s nucleus (Gibson et al., 1988). Varicose CGRP peptide-positive fibers are observed in spinal laminae VII–IX and in association with the neurons of Onuf’s, together with CPON-positive fibers that appear numerous around Onuf’s cells. Additionally, some neurons of Onuf’s nucleus are innervated by neurokinin-like-immunoreactive fibers. A strikingly dense innervation of somatostatin-positive fibers is observed, whereas no Thyrotropin Releasing Hormone (TRH)-positive elements are visible around Onuf’s neurons. Finally, small numbers of Vasoactive Intestinal Polypeptide-immunoreactive fibers are found associated with Onuf’s nucleus (Gibson et al., 1988; Katagiri et al., 1988), and dopamine beta-hydroxylase (DBH)-immunoreactive fibers were described in rat Onuf’s nucleus by Kojima et al. (1985).

Thus, since innervation, peptide pattern distribution and neuronal integrity are lost in somatic MN diseases (such as ALS), and conversely spared in Onuf’s nuclei, it could be speculated that the pattern of innervation and peptide expression contributes to the maintenance of Onuf’s neural integrity (Gibson et al., 1988).

Similarly, both muscle fiber composition and innervation could influence the selective vulnerability of MNs. As known, muscle fibers can be divided into fast-twitch glycolytic (type IIb), fast-twitch oxidative (type IIa), and slow-twitch oxidative (type I) groups. Histochemical and morphometrical analyses on human bulbospongiosus and ischiocavernosus muscles (innervated by Onuf’s MNs) revealed a high variability of the distribution of the two fiber types, with type I fibers abundantly expressed, especially in ischiocavernosus muscle (Ravnik and Sirca, 1993). Moreover, the oxidative activity was higher in type I fibers, which notably are usually smaller in diameter than type II (Schrøder and Reske-Nielsen, 1983; Ravnik and Sirca, 1993).

Finally, also non-cell-autonomous events could contribute to the progression of MN diseases. For instance, astrocytes in sporadic ALS samples appear harmful to somatic MN survival (Haidet-Phillips et al., 2011), whereas Onuf’s MNs and oculomotor nuclei seem less vulnerable to toxic astrocytes. Indeed, it is still unclear if either: (i) these MNs are more resistant to neuroinflammation *per se*; or (ii) the astrogliosis is less intense around these resistant nuclei; or (iii) astrocytes surrounding Onuf’s nucleus differently react to degeneration (Nijssen et al., 2017).

## Recent Insights From Genomics and Proteomics

The differential vulnerability of Onuf’s neurons has been initially attributed to their cytoarchitecture, connectivity, and metabolic rate (Onuf, 1899; Schrøder, 1981; Gibson et al., 1988; Shaw and Eggett, 2000). Only recently, in the XXI century, it has been evaluated by more sophisticated genetic and molecular analyses (Kaplan et al., 2014; Allodi et al., 2019).

The first evidence comes from studies on the ontogeny of MNs, which is led by differential expression of transcription factors guiding neuronal differentiation and diversification during embryogenesis (Cave and Sockanathan, 2018). Concerning the Onuf’s neurons, the LIM homeodomain protein Islet-1 seems involved in their embryonic generation: indeed, in the murine spinal cord, at embryonic days 13.5–15.5, the highest expression of Islet-1 is reported in MNs innervating EUS and ischiocavernosus muscles, and to a lesser extent in the sacral parasympathetic preganglionic neurons. These findings suggest that Onuf’s neuron development can be guided in a distinct transcriptional way compared to other MN groups at the prenatal stages (Han et al., 2009).

Valuable insights into the resistant/vulnerable nature of specific MN pool also came from another cell population, i.e., oculomotor neurons, which share many commonalities with Onuf’s neurons and are similarly spared in some pathologies (as ALS and SMA). Pioneering transcriptome studies revealed hundreds of genes differentially enriched in the oculomotor neurons, including insulin-like growth factor II and guanine deaminase, playing a pivotal neuroprotective function against glutamate-induced neurotoxicity (Hedlund et al., 2010). Accordingly, in 2013, microarray analysis demonstrated that human oculomotor neurons show a distinct transcriptional profile, compared to somatic spinal MNs. Indeed, approximately 1,700 genes (mainly related to synaptic transmission, ubiquitin-dependent proteolysis, mitochondrial activity, and transcriptional regulation) resulted differentially expressed: in particular, the reduced susceptibility to excitotoxicity of these neurons seems to play an important role in assuring resistance to neurodegeneration (Brockington et al., 2013).

More recently, Kaplan et al. (2014) studied in ALS mice the gene expression profile of both Onuf’s and oculomotor nuclei, compared to vulnerable spinal MNs. The authors performed a microarray profiling of MNs isolated from P7 WT or ALS mice, to identify the selective genetic signature of Onuf’s and oculomotor nuclei (resistant in ALS) and lumbar spinal MNs (vulnerable; Kaplan et al., 2014). The authors found 18 genes more than ten-folds differentially expressed in vulnerable than in resistant MNs and identified 14 of these genes expressed only in the vulnerable ones. Moreover, they showed that Semaphorin3a (SEMA3A), Glutamate decarboxylase 2 (GAD2) and potassium voltage-gated channel subfamily J member 5 (KCNJ5) genes were enriched in oculomotor and Onuf’s nuclei, whereas Natriuretic Peptide Receptor 3 (NRP3), TRH Receptor (TRHR), Neurotensin (NTS), Egl-9 Family Hypoxia Inducible Factor 3 (EGLN3), 17β-Hydroxysteroid dehydrogenase 2 (HSD17B2) and MMP9 (matrix metallopeptidase 9) genes were depleted in the resistant population of Onuf’s and oculomotor MNs. In particular, MMP9 was strongly expressed by the majority of cranial and spinal MNs, and undetectable in Onuf’s nucleus. Thus, MMP9 can represent a prospective marker for ALS-affected MNs, also because its high expression can be detected even in the neonatal spinal cord, before the earliest known functional changes occurring in ALS (Kaplan et al., 2014; Nijssen et al., 2017). Indeed, reduction or deletion of MMP9 delay muscle denervation and extend the survival of transgenic mice (Kaplan et al., 2014). The extracellular matrix protein osteopontin (OPN) is selectively expressed by ALS-resistant MN pools, and the difference in OPN/MMP9 expression profiles in MN populations could partially explain the selective vulnerability of cells in neurodegeneration. OPN is involved in the second wave of neurodegeneration in ALS disease by upregulating MMP9 through αvβ3 integrin (Morisaki et al., 2016).

Recently, Allodi et al. (2019) isolated Onuf’s MNs from human post-mortem tissue, together with oculomotor and spinal (from cervical and lumbar segments) MNs, and analyzed their marker expression by spatial transcriptomic method LCM-seq. While all MN groups expressed high levels of neurofilaments (NFs) and Vesicular acetylcholine transporter (VACHT), Onuf’s MNs selectively and uniquely expressed 349 up- and 572-downregulated expressed genes (DEGs). Interestingly, gene ontology analysis revealed that those genes enriched in Onuf’s neurons are involved in trans-synaptic and anterograde synaptic signaling, ATP biosynthesis processes, regulation of neurotransmitter levels, regulation of exocytosis and synaptic vesicle localization. The study also revealed that oculomotor and Onuf’s MNs mainly showed distinct gene pattern regulation, even if some common pathways were identified. Indeed, the authors found that Onuf’s and oculomotor MNs shared 214 DEGs, of which 142 were downregulated and 58 upregulated compared to spinal MNs, and 14 genes were regulated in opposite direction in the two nuclei. The common pathways could underlie the joint resilience of Onuf’s and oculomotor nuclei in spinal neurodegenerative diseases. For instance, MIF (macrophage migration inhibitory factor), which has been demonstrated to be neuroprotective in ALS (Shvil et al., 2018), showed a high expression both in Onuf’s and oculomotor nuclei compared to the other spinal MNs (Allodi et al., 2019). Instead, among the 14 opposite-regulated genes, some are involved in neurogenesis and apoptosis regulation (Ephrin type-A receptor 7, sparc/osteonectin1), in the regulation of proteins associated with the cytoskeleton (PDZ and LIM Domain 1), in the degradation of transmembrane proteins (Ras-related protein RAB12), in the stabilization of cell membrane potential and maintenance of intracellular pH (CLIC1) and in receptor assembly and activity (e.g., Progestin and AdipoQ Receptor Family Member 8, gamma-aminobutyric acid type A receptor alpha1 subunit, Gamma-Aminobutyric Acid Type A Receptor Subunit β1; Allodi et al., 2019).

These studies demonstrated that gene and/or protein expression analysis of MNs that show differential susceptibility to degeneration can be used to point out candidates to preserve vulnerable MNs. The studies highlight that neuronal vulnerability in the spinal cord could be regulated by the lack of certain beneficial factors and proper innervation, as well as by the increase in susceptibility to noxious molecules (Nijssen et al., 2017).

## Involvement of Onuf’s Nucleus in Diseases

**Figure 4:**
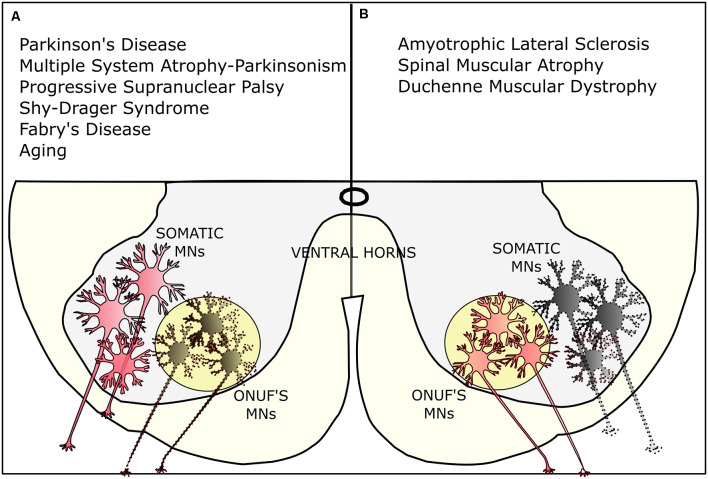
Selective vulnerability of Onuf’s MNs. **(A)** Onuf’s nucleus is affected in several neurodegenerative diseases, such as Parkinson’s disease (PD) and multiple system atrophy (MSA) Parkinsonism, the progressive supranuclear palsy (PSP), the Shy-Drager-Syndrome, and Fabry’s disease. Onuf’s MNs are also degenerating in aging-related pathologies, leading, for instance, to urinary incontinence. **(B)** On the contrary, Onuf’s nucleus is spared in certain neurodegenerative diseases where, conversely, somatic MNs are degenerating: for instance, Onuf’s MNs are preserved in amyotrophic lateral sclerosis (ALS), spinal muscular atrophy (SMA), and duchenne muscular dystrophy (DMD). Dotted lines represent dying/affected MNs.

For its peculiarities, Onuf’s nucleus is involved or affected in aging-related pathologies as well as several traumatic and neurodegenerative disorders (as PD and SDS), whereas it is spared in other diseases (ALS, SMA, DMD) that we describe below (Figure 4). Although extremely important in pediatrics, here we will not deal with the Onuf’s nucleus development and related defects.

### Urinary Incontinence and Aging

Micturition is allowed by the contraction of the detrusor muscle of the bladder and by the coordinated relaxation of two urethral sphincters: the external sphincter (also known as urogenital sphincter) and the internal sphincter (a.k.a. trigone sphincter), that are respectively innervated by voluntary and autonomic control (reviewed by Jung et al., 2012). Accordingly, the urethra wall receives the innervation by both autonomic and somatic nervous system: (i) the sympathetic and parasympathetic innervation is dedicated to the longitudinal and circular smooth muscle layers in the urethra, whereas (ii) the somatic innervation (involving the Onuf’s nucleus) through the pudendal nerve is dedicated to the striated muscle rhabdosphincter (Thor, 2004).

The cerebral cortex, POSC, and the thoracic, lumbar, and sacral spinal regions control the lower urinary tract. In particular, as described above, the prefrontal cortex, through the medial OFC with its projections to the PAG, plays an important role in micturition (Holstege, 2016).

The degeneration of the above-mentioned pathways leads to urge-urinary incontinence (UI) in patients, due to altered communication between cortex, PAG-POSC, and Onuf’s nucleus. In this case, the PAG activates micturition function based only on incoming information from the pelvic organs (without the cortical control): thus, only a relatively small amount of urine is sufficient to activate the pathway, leading to uncontrolled micturition (Holstege, 2016). Aging, obesity, multiple labors, and prostatic surgery could lead to UI. Additionally, stress urinary incontinence (SUI) is characterized by the leakage of urine because of an effort, a physical exercise, or sneezing/coughing (Haylen et al., 2010).

The current treatments for SUI involve behavioral therapy, pelvic floor muscle training, pharmacological treatment, and surgery (including urethral bulking injections; Padmanabhan and Dmochowski, 2014). However, the surgical approach can be associated with complications or poor effectiveness. Besides, also stem cell therapy has been evaluated as a promising treatment (Gunetti et al., 2012; Gallo et al., 2019).

It has been described that serotonin and noradrenaline are involved in the central neural control of the lower urinary tract function: the pudendal somatic motor nucleus of the spinal cord is densely innervated by serotonin (5-HT) and norepinephrine (NE) terminals. 5-HT is released from the raphe nuclei in the brainstem and binds to different receptors (5-HT1A, 5-HT2A, 5-HT2C, 5-HT3, and 5-HT7; as reviewed by Panicker, 2019). Indeed, Xu et al. (2007) described in the rat Onuf’s nucleus the presence of several groups of MNs innervating different pelvic striated muscles: these neurons differentially express 5-HT2AR and 5-HT5AR. In particular, 5-HT2ARs are mainly localized in MNs innervating the ischiocavernosus muscle, whereas 5-HT5ARs are specifically expressed in neurons innervating the external urethral sphincter and bulbospongiosus muscles (Xu et al., 2007). Serotonin and noradrenaline modulate the effect of the neurotransmitter glutamate, which in turn activates the neurons in the Onuf’s nucleus, resulting in the contraction of the sphincters (Michel and Peters, 2004; Panicker, 2019). In general, serotonergic receptor agonists exert a pro-continence effect by suppressing parasympathetic activity and increasing somatic and sympathetic activity in the lower urinary tract (Michel and Peters, 2004). Drugs, as duloxetine, can act by blocking the 5-HT and NE reuptake and by increasing the receptor activation at the presynaptic neuron in Onuf‘s nucleus of the sacral spinal cord: as a consequence, the sphincter MN activity is increased (Jost and Marsalek, 2004) and the control of urine storage/micturition improved (Thor, 2004).

Similarly, aging (characterized by changes in central monoaminergic neurons) can cause urinary dysfunction. Quantitative image analysis revealed significant age-associated declines in the innervation of the intermediolateral nucleus, sacral parasympathetic nucleus, dorsal gray commissure, and Onuf’s nucleus (Ranson et al., 2003). Studies on aged rats revealed that mean and maximal micturition volumes are significantly larger compared to young animals (Chun et al., 1988), due to changes in the innervation of the bladder and central control of micturition. In 2010, Zhao and colleagues (Zhao et al., 2010) confirmed that “in old rats, the bladder capacity and the post-void residual urine are higher, and responses to muscarinic receptor stimulation and electrical field stimulation are lower than in young rats.” These findings correlate with observations from aged human bladders, in whom decreased detrusor contractility and detrusor overactivity are among the most conspicuous age-related changes in bladder function (Hald and Horn, 1998).

Indeed, urinary incontinence is also reported in the case of Alzheimer’s disease (AD) and dementia with Lewy bodies, due to detrusor overactivity (Del-Ser et al., 1996; Ransmayr et al., 2008). This is probably due to pathologic changes in the frontal cortex and substantia nigra (Attems et al., 2007; Ransmayr et al., 2008); moreover, supra-pontine lesions resulting from AD can affect the cortical inhibitory control of POSC (Yokoyama et al., 2002).

### Spinal Cord Injury

A spinal cord injury (SCI) induces damage at the level of the spinal cord that causes temporary or permanent changes in neuronal function and innervation. Symptoms include loss of sensations, paralysis, or autonomic dysregulation, below the level of the injury (Eckert and Martin, 2017).

Therefore, SCI can also affect Onuf’s MNs and their innervation of the EUS muscle: indeed, in case of trauma above the lumbosacral level, EUS fails to relax while the bladder is contracting, leading to voiding impediment, urine retention, bladder pressure, finally causing renal failure (Weld et al., 2000; de Groat and Yoshimura, 2006; Ni et al., 2019). More in detail, after SCI, the serotoninergic nerve fibers start to degenerate below the level of injury (Ni et al., 2019); then, a significant increase in the number of 5-HT7 receptors (5-HT7Rs) in the Onuf’s nucleus has been observed in SCI rat models, together with dysfunctions in urethra opening and closing (Gang et al., 2014; Ni et al., 2019; Panicker, 2019). By injecting intrathecally in SCI rats a selective agonist for 5-HT7R, Ni and colleagues obtained a significant dose-dependent increase in micturition volume and voiding efficiency (Ni et al., 2019). The change in the expression of serotonin receptors after SCI must be confirmed in human Onuf’s cells, but these recent findings suggest the 5-HT7R could be a possible target for therapeutic interventions.

Moreover, in men, Onuf’s neurons also innervate the striated perineal muscles attached to the base of the penis (Breedlove and Arnold, 1980), thus being involved in ejaculation functions and subsequent sexual behavior. SCI at this spinal level frequently leads to sexual dysfunction in men, including erectile and ejaculation disorders (Chéhensse et al., 2013; Sakamoto, 2014; Krassioukov and Elliott, 2017). In patients with complete injuries of S2-S4 segments, a rhythmic forceful ejaculation is precluded; moreover, urine leaks due to urinary sphincter hypotonia or atonia are often reported. These data support the idea that the parasympathetic and somatic centers located in S2-S4 segments control the emission and expulsion phases of ejaculation in men (Chéhensse et al., 2013): in particular, Onuf’s nucleus participates in ejaculation, innervating the bulbospongiosus muscle and consequently favoring the semen progression from the bulbar to the penile urethra (Gerstenberg et al., 1990).

Moreover, the nervous pathway for the voluntary control of fecal continence and defecation also involves the Onuf’s nucleus. Defecatory disorders (e.g., difficulties in defecation, fecal incontinence, and chronic constipation) are common severe problems in SCI patients and are generally attributed to a dysfunctional control of peristalsis and colorectal motility by the CNS (Lynch and Frizelle, 2006). In SCI condition, the interactions between the somatic and autonomic components are compromised: indeed, spinal cord trauma leads to a failure of supraspinal inputs modulating the lumbosacral defecation centers and the Onuf’s nucleus, affecting voluntary motor functions and uncoordinated anorectal reflexes (Guertin, 2016). In particular, descending fibers from the brainstem lateral cell group make synapses on those Onuf’s MNs that supply the external anal sphincter (Callaghan et al., 2018). To preserve continence, afferents to the rectum start contractions of the external anal sphincter through spinal reflexes, and consistent with the presence of local reflex control of the external sphincter, connections are made to Onuf’s nucleus from the spinal afferents and the sacral regions of the defecation center. Some of these local inputs to Onuf’s nucleus are inhibitory (Beckel and Holstege, 2011; Callaghan et al., 2018). Moreover, there is some evidence in humans that spinal circuits can influence the external sphincter independently of brain descendent pathways (Broens et al., 2013).

### Parkinson’s Disease and Parkinsonian-Like Syndromes

PD is a severe neurodegenerative pathology due to the progressive loss of dopaminergic neurons in the substantia nigra, determining tremors, bradykinesia, rigidity, impaired posture and balance (Beitz, 2014).

Among the commonest non-motor symptoms in PD, urinary urgency, increased daytime frequency, and nocturia is reported (Sakakibara et al., 2012). These bladder symptoms are probably a consequence of the cell death in the substantia nigra causing progressive loss of the D1 receptor-mediated tonic inhibition of the micturition reflex, which in turn leads to detrusor muscle hyperactivity (Winge and Fowler, 2006). Indeed, the disruption of the suprapontine circuitry eliminates the inhibitory control over the POSC, decreasing bladder capacity, and causing detrusor overactivity (Fowler et al., 2008).

Alterations at the Onuf’s nucleus level have been clinically reported for the first time in 2008, in a 59 year-old PD patient (O’Sullivan et al., 2008): in this patient, deterioration into a hypo-compliant bladder, with subsequent evidence of obstructions and bladder instability, worsened together with PD progression. The analysis of the patient nervous tissue revealed the accumulation of a-synuclein-positive Lewy bodies not only in the substantia nigra, the cortex, and brainstem but also in the spinal cord. Indeed, scattered Lewy neurites and Lewy bodies were found in the gray matter of the intermediolateral columns of the spinal cord, at the thoracic and sacral levels, including the Onuf’s nucleus. Generally, PD patients do not show severe sphincter abnormalities in the early stages of the disease, whereas some cases of neurogenic changes of external anal sphincter muscle have been observed in advanced stages of PD (Libelius and Johansson, 2000; O’Sullivan et al., 2008).

The neurodegeneration of Onuf’s nucleus is one of the pathological landmarks found in MSA Parkinsonism (MSA-P) disease. Indeed, many cases of MSA-P are characterized by the almost complete neuronal degeneration in Onuf’s nucleus, associated with progressive loss in urinary function: Onuf’s impairment is considered as responsible for denervation of the urethral sphincter and external anal muscles observed in MSA-P (Yamamoto et al., 2005; O’Sullivan et al., 2008). The involvement of Onuf’s nucleus in the progression of the disease seems to be time-dependent, in the early phases of the disease, no signs of neurogenic changes are detected, while pathological signs become progressively evident with the increasing incontinence (storage and voiding disorders) and in detrusor muscle over-activity (Yamamoto et al., 2005, 2014).

Autonomic urinary dysfunction has been described also in patients with Progressive Supranuclear Palsy (PSP), a rare brain Parkinson-like disorder that causes progressive problems in gait and balance (Yamamoto et al., 2016). In PSP patients, urinary storage dysfunctions are not different from that of PD and MSA-P, whereas urinary voiding dysfunction in PSP results more severe than in PD, while milder than in MSA-P (Yamamoto et al., 2016). The Onuf’s nucleus degeneration in PSP remains to be clarified. Three cases of Onuf’s neuronal loss have been reported by Scaravilli et al. (2000) in PSP patients with clinical evidence of sphincter disturbances. In these patients, together with neuropathological changes in cortical, subcortical, and autonomic centers already described for PSP (Kasashima and Oda, 2003; Halliday et al., 2005), neurofibrillary tangles were observed across the spinal cord, through the intermediolateral columns and Onuf’s nuclei. Besides, neuropil threads and glial inclusions were also observed in Onuf’s nucleus region (Scaravilli et al., 2000). Moreover, the nucleus displayed a severe cell loss: morphological and morphometrical analyses showed a reduction of more than 50% in the number of MNs, together with the reduction in cell diameters compared to healthy conditions (Scaravilli et al., 2000).

Conversely, other works based on quantitative motor unit potential (MUP) analysis did not reveal any alteration in Onuf’s nucleus related to bladder dysfunction in PSP patients (Yamamoto et al., 2016). They found that the mean value of MUPs in PSP patients showed a duration (less than 10 ms) that does not indicate neurogenic changes in Onuf’s nucleus. MUP duration reflects the progression of frontal cortex degeneration in PSP patients, but the neurodegeneration in Onuf’s nucleus might be occurring in parallel (Yamamoto et al., 2016). However, the rarity of the disease and the limitation in PSP (spinal) sample availability might constitute a restriction in PSP pathophysiological studies and large cohorts of patients are needed to better unravel the involvement of Onuf’s nucleus.

### Shy-Drager Syndrome and Fabry’s Disease

SDS is a movement disorder that can be referred to as a PD plus MSA. SDS patients show rigidity and bradykinesia as primary extrapyramidal symptoms, together with autonomic nervous system dysfunctions which lead to much of the disability seen in this disorder. Indeed urinary incontinence, syncope, constipation, fecal incontinence, cardiac arrhythmias as well as other symptoms are the result of the widespread pathological changes occurring in multiple areas of the central and autonomic nervous system in SDS (Hodder, 1997). Since early stages, SDS patients show loss of sphincter tone as well as fecal and urinary incontinence compatible with chronic denervation (Shy and Drager, 1960; Sung et al., 1979).

A general alteration at the spinal cord level is observed: sacral cross-sections are smaller in size than the healthy controls and the anterior median fissure appears slightly widened at the levels Sl-S3. Moreover, MNs in the ventral horns are decreased in number: the posteromedial and posterolateral groups are the most highly affected compared to the anteromedial and anterolateral groups. Onuf’s nucleus appears atrophied and without the peculiar paleness of the neuropil. Indeed, histological analysis revealed that in many SDS cases the neurons of Onuf’s nucleus are almost entirely lost (Sung et al., 1979; Mannen et al., 1982). Together with this selective neuronal loss, SDS is also characterized by marked abnormalities in the external anal sphincter muscle, characterized by thickness reduction, increase in sarcolemmal nuclei number and in endomysial connective tissue (as signs of neurogenic muscular atrophy; Mannen, 1991).

Alterations in Onuf’s nucleus have been also observed in other pathologies involving multiple systems, with major dysfunctions in the autonomic nervous system. For instance, Fabry’s Disease (FD) is an X-linked lysosomal storage disorder due to a deficiency in α-galactosidase-A activity; this leads to the subsequent accumulation in various tissue and organs of the globotriaosylceramide (Gb3) substrate, which contributes to disease pathology (Sung, 1979; Khanna et al., 2010). The nervous system is particularly affected, and interestingly, neuronal lipid storage is limited to certain nuclei, such as the amygdala, hippocampus, hypothalamus, substantia nigra, reticular formation of the brainstem, ambiguus and dorsal vagal nuclei of the medulla oblongata and the lateral horns of the spinal cord (Sung, 1979). Indeed, it was observed that autonomic centers throughout the neuro-axis show a predilection for lipid storage, thus representing a landmark marker of FS disease: due to these lipid depots, Onuf’s neurons acquire a typical “ballooned” morphology (Sung, 1979). Interestingly, the accumulation of Lewy bodies was recently observed in many areas of the brain and in the spinal cord lamina IX of an FS patient (Del Tredici et al., 2020): this suggests that a more detailed analysis of human FD spinal cord might reveal the presence of pathological inclusions also in the Onuf’s nucleus.

### Amyotrophic Lateral Sclerosis and Spinal Muscular Atrophy

ALS is a neurodegenerative disease targeting the upper and lower somatic MNs, which innervate voluntary muscles, leading to muscle atrophy and weakness of skeletal muscles. MNs of the autonomic system is less affected (Piccione et al., 2015). Approximately 10% of ALS cases are familial (fALS), while 90% are sporadic (sALS). Mutations have been identified in more than 20 genes (e.g., Superoxide dismutase 1) and contribute to the two-thirds of fALS and for about 10% of sALS (Chia et al., 2018). Moreover, many complex and correlated pathological mechanisms (such as mitochondrial dysfunction, protein aggregation, excitotoxicity, and inflammation) have been identified as contributors to MN malfunction and death (Robberecht and Philips, 2013).

Interestingly, subtypes of affected spinal MNs display a gradient of vulnerability, with fast-twitch fast-fatigable (FF) MNs degenerating before slow MNs, while specific MN pools are more resistant to neurodegeneration, such as the oculomotor neurons and neurons of the Onuf’s nuclei (Nijssen et al., 2017). Consequently, muscle denervation occurs early on type IIb muscle fibers, mainly innervated by FF MNs, followed by a second wave of denervation affecting the type IIa muscles, due to the degeneration of fatigue-resistant MNs (Frey et al., 2000; Kaplan et al., 2014). Instead, the slow MNs are resistant almost until the disease end-stage, still providing the innervation of type I fibers (Rochat et al., 2016). Muscles innervated by Onuf’s MNs might result in more resistance to denervation due to the higher percentage of small type I muscle fibers, compared to other skeletal muscles; the presence of slow MNs in Onuf’s nucleus could partially explain Onuf’s neuron resilience in some neurodegenerative diseases.

Indeed, given the peculiar characteristics of Onuf’s and oculomotor MNs described above (up/down regulation of specific genes compared to spinal MNs as showed in Kaplan et al., 2014; Allodi et al., 2019), it is not surprising that these neurons respond differently in ALS compared to the other MNs, remaining resistant to degeneration until the later stages of the disease. For instance, as stated, it has been demonstrated that MMP9 is highly expressed by ALS-affected spinal MNs, whereas its expression is absent in Onuf’s neurons; on the contrary, the protein OPN is selectively expressed by ALS-resistant pool, while downregulated in vulnerable MNs (Kaplan et al., 2014).

Cytoskeletal abnormalities in Onuf’s nucleus have been rarely addressed in ALS patients: sporadic atrophic neurons with evident central chromatolysis or cells showing conglomerate inclusions (e.g., spheroids, anti-NF positive inclusions, ubiquitin-reactive inclusions, Bunina bodies) were infrequently detected (Okamoto et al., 1991; Bergmann et al., 1995; Kihira et al., 1997). By using correlative morphometry analysis, Takeda et al. (2014) showed a decreased cell circularity index at the early phase also in Onuf’s nucleus, although neuronal perimeter and area of Onuf’s neurons remained stable even in advanced phases of disease.

Given that in ALS Onuf’s nucleus is only partially vulnerable to the ALS process and the degree of degeneration differs from that observed in other MNs, the involvement of Onuf’s nucleus might be slowed due to characteristics specific to this nucleus, including its physicochemical and molecular properties.

Interestingly, also in SMA, the autosomal recessive MN disease representing the most common genetic cause of infant mortality, patients retain both normal eye movements and external sphincter continence. Indeed, oculomotor and Onuf’s nuclei are preserved in SMA as well as in ALS (Sumner et al., 2017). Thus, the lack of Survival Motor Neuron (SMN) protein, which is the specific hallmark of MNs in SMA pathology, could only partially affect Onuf’s cells. Deep transcriptomic analysis in SMA tissues might reveal opposite-expressed genes between Onuf’s and the spinal MNs, eventually related to SMN function.

The most severe form of SMA (SMA type I) is also known as Werdnig-Hoffman disease (WHD). In addition to SMN lack, it is characterized by the presence of glial bundles in the anterior nerve roots, with relative preservation of the phrenic MNs and diaphragm, and the presence of ballooned neurons immunohistochemically positive for phosphorylated NF (Kuroiwa et al., 1997). Although these lesions mainly affect the motor system, some studies also reported the involvement of the cerebellum, thalamus, and other regions (Norman and Kay, 1965). In the spinal cord, the loss of MNs in the thoracolumbar regions is accompanied by mild fibrous astrogliosis and loss of myelin. These characteristics appear more striking in the lumbar segments: here, the neurons of the intermediolateral and Clarke’s nuclei are relatively well preserved. Instead, in the sacral segments, the neurodegeneration together with increased astrogliosis is diffusely observed, but Onuf’s nucleus neurons appear spared (Kuroiwa et al., 1997). Only in rare cases (about 5%), Onuf’s neurons showed central chromatolysis, but no differences in the number and mean cell diameter were observed between WHD and control tissues (Kumagai and Hashizume, 1982).

Similarly, in the case of Spinal Bulbar Muscular Atrophy (SBMA), an X-linked disorder caused by expansion of a CAG trinucleotide repeat in the androgen receptor (AR) gene, the oculomotor and Onuf’s nuclei resist to neurodegeneration (Sobue et al., 1981). It was initially proposed that the sparing of these neurons was due to lack of ARs (Sar and Stumpf, 1977), but further studies revealed a high expression of AR protein in Onuf’s nucleus (Merry, 2001), ruling out this possibility.

### Duchenne Muscular Dystrophy

The DMD is an X-linked neuromuscular disorder caused by mutations in the dystrophin gene. The absence of dystrophin protein leads to progressive muscular weakness and wasting, causing degeneration of both skeletal and cardiac muscles (Falzarano et al., 2015). Onuf’s nucleus appears spared in DMD patients (Iwata and Hirano, 1978): indeed, in DMD bladder and sphincter dysfunction are rarely reported, even in advanced disease stages. As already observed in other diseases (e.g., ALS), the sparing of neurons in the Onuf’s nucleus seems to limit the fiber loss in the external urethral sphincter and, in general, in the pelvic floor (Pullen and Martin, 1995). Some DMD patients show a detrusor-sphincter dyssynergia, but concomitant with other dysfunctions, such as cerebral palsy and paraplegia following scoliosis surgery (Dyro and Bauer, 1990).

## Concluding Remarks

Onuf’s nucleus is anatomically and physiologically intriguing. Its MNs are histologically comparable to the other somatic MNs (Onuf, 1899; Pullen et al., 1997) and innervate striated voluntary muscles. However, curiously, Onuf neurons also show some autonomic-like features as, for instance, they receive presynaptic connections from the midbrain nuclei that project both to Onuf’s and autonomic preganglionic parasympathetic neurons (Pullen et al., 1997). Moreover, a strong peptidergic presynaptic input associates Onuf’s nucleus with autonomic neurons (Kawatani et al., 1986; Tashiro et al., 1989a; Pullen et al., 1997).

As described above, these peculiarities make Onuf’s nucleus alternatively vulnerable or resistant to neurodegeneration in different neurological diseases. Through the years, progressively more sophisticated techniques revealed that a specific morphological, biochemical, metabolic and genetic signature could be responsible for their differential vulnerability/resistance to degeneration (Onuf, 1899; Schrøder, 1981; Gibson et al., 1988; Shaw and Eggett, 2000; Kaplan et al., 2014; Allodi et al., 2019).

Altogether, these studies pave the way to design new therapeutic strategies for counteracting neurodegeneration. However, as highlighted, the majority of recent data refer to ALS. Therefore, additional analyses are still needed to better explain the resilience/vulnerability of Onuf’s neurons in other pathologies, seen the heterogeneity in the disease etiology and onset, in the age of patients, in the distribution of vulnerable motor pools (e.g., diaphragmatic function is preserved in SMA and affected in ALS) and molecular targets.

## Author Contributions

MB: conceptualization and draft preparation. RS and MB: writing, figures, and revision. MB and AV: final revision.

## Conflict of Interest

The authors declare that the research was conducted in the absence of any commercial or financial relationships that could be construed as a potential conflict of interest.
